# Use of Integrative Medicine in the United States Military Health System

**DOI:** 10.1155/2017/9529257

**Published:** 2017-06-13

**Authors:** Cathaleen Madsen, Megan Vaughan, Tracey Pérez Koehlmoos

**Affiliations:** ^1^Uniformed Services University of the Health Sciences, Bethesda, MD, USA; ^2^Henry M. Jackson Foundation for the Advancement of Military Medicine, Bethesda, MD, USA; ^3^Defense and Veterans Center for Integrative Pain Management, Rockville, MD, USA

## Abstract

Integrative medicine (IM) is a model of care which uses both conventional and nonconventional therapies in a “whole person” approach to achieve optimum mental, physical, emotional, spiritual, and environmental health, and is increasingly popular among patients and providers seeking to relieve chronic or multifactorial conditions. The US Department of Defense (DoD) shows particular interest in and usage of IM for managing chronic conditions including the signature “polytrauma triad” of chronic pain, traumatic brain injury (TBI), and posttraumatic stress disorder (PTSD) among its beneficiaries in the Military Health System (MHS). These modalities range from conventional nondrug, nonsurgical options such as cognitive-behavioral therapy to nonconventional options such as acupuncture, chiropractic, and mind-body techniques. These are of particular interest for their potential to relieve symptoms without relying on opiates, which impair performance and show high potential for abuse while often failing to provide full relief. This review describes the use of IM in the MHS, including definitions of the model, common therapies and potential for use, and controversy surrounding the practice. More research is needed to build a comprehensive usage analysis, which in turn will inform sound clinical and financial practice for the MHS and its beneficiaries.

## 1. Introduction

Integrative medicine (IM) is a current healthcare paradigm which promotes a “whole person” approach to health through coordinated use of appropriate therapies originating both inside and outside of conventional medicine. Though often conflated with complementary and alternative medicine (CAM), changes in the definition of these terms make this conflation technically inaccurate. The American Board of Physician Specialties describes IM according to five areas: patient-provider partnership; consideration of all factors, including mental, physical, and spiritual, in addressing health, wellness, and disease; facilitation of the body's innate healing response through use of conventional and nonconventional methods; the use of less-invasive and less-harmful treatments whenever possible to treat the whole patient as well as the disease; and the ideal of medicine as science-based, inquiry-driven, and willing to give critical consideration to new paradigms [1]. The National Center for Complementary and Integrative Health (NCCIH) at the National Institutes of Health (NIH) defines nonconventional modalities as alternative when used in place of mainstream therapies, complementary when used as adjunct to mainstream therapies, and integrative when used as part of a coordinated system of care [2]. However, classification of therapies varies widely between sources and depends heavily on usage. Additionally, the lines between IM and conventional medicine are increasingly blurred, as nonconventional therapies such as acupuncture and chiropractic are now taught in mainstream American medical schools [3] and familiar nondrug, nonsurgical options such as cognitive-behavioral therapy are offered under the umbrella of IM [4].

The NCCIH estimates that 30% of adults and 12% of children in the United States use nonconventional therapies [2]. This use often corresponds with multifactorial or chronic illnesses, which are notoriously difficult to treat despite the involvement of multiple specialists [5, 6], and greater usage of nonconventional procedures often corresponds with greater number of comorbidities [7]. Accordingly, the Department of Defense (DoD) has shown increasing interest in and usage of IM for managing chronic conditions within the Military Health System (MHS), particularly the signature “polytrauma triad” [8] of chronic pain, traumatic brain injury (TBI), and posttraumatic stress disorder (PTSD) among wounded warriors [9]. A nationwide study of veterans (*n* = 613,391) found a three-year prevalence of 9.6% for TBI, 29.3% for PTSD, and 40.2% for pain, with 6.0% exhibiting the full polytrauma triad [8]. Symptoms of each disorder can overlap and reinforce the others, particularly in the case of chronic pain and PTSD, posing a diagnostic and therapeutic challenge [10, 11]. Chronic pain itself is a particular problem, as conventional treatment relies on opioids which impair functionality, have potential for abuse, and often fail to relieve symptoms fully [12]. This leads to an overall decrease in readiness, a significant concern for a military population [13–15].

A 2004 survey of military members and families found 81% who reported using nonconventional modalities and 69% of respondents desiring more IM options within the MHS [16]. Some research shows that military members use nonconventional therapies at a higher rate than do civilians [14], even if they must be obtained outside the MHS. Therefore, promotion of IM within the MHS reflects an effort to recapture these patients by providing care which many have already found effective [7, 14, 17], as well as to offer coordinated nondrug, nonsurgical options to new patients.

This review summarizes the available literature to describe the IM therapies being used in the MHS, including the conditions for which they are sought and the evidence base for their use. This research aims to inform decision-making for integration of nondrug, nonsurgical options into coordinated care for military members and their beneficiaries.

## 2. Methods

The foundations of this paper were three surveys of IM in the MHS, reflecting practices in 2005 and 2009 [18], 2013 [4], and 2010–2015 [19]. These included a total of 23 therapies with some overlap, which were combined where thematically appropriate (e.g., meditation, mindfulness, and guided imagery) to give a total of 15 categorical modalities. Integrative therapies not covered by one of these lists were excluded from further discussion as prevalence in the MHS could not be determined from published literature. Determination of therapies as “nonconventional” was according to the Cochrane Collaboration's list of 51 therapies considered to be “alternative” or “complementary” [20].

Supporting evidence for therapies, including indications for use, mechanism of action, and effectiveness where available, was sourced through publicly available reports of the government, military, and nongovernmental research organizations and peer-reviewed literature published in or after 2000. This was the year of the Presidential directive establishing the White House Commission on Complementary and Alternative Medicine, marking an official change in focus from restricting access to enabling use of nonconventional therapies [21].

Additional clarification, such as definition of terms, licensing information, or coverage of treatments, was obtained from official websites of organizational bodies (e.g., American Board of Physician Specialities, American Association of Naturopathic Medical Colleges, TRICARE).

## 3. Results

### 3.1. Types of Therapies

Three major surveys have assessed use of IM within the MHS. This healthcare system serves 9.4 million military members, beneficiaries, and retirees at Military Treatment Facilities (MTFs) including 55 hospitals and 373 military medical clinics [22, 23]. The earliest surveys, by Petri and Delgado in 2005 and 2009, found 19 modalities collectively available at 14 MTFs. However, only 15 modalities are reported by name [18]. The most widely offered modalities in 2005 were chiropractic, transcutaneous electrical nerve stimulation (TENS), nutritional counseling, and meditative behavioral techniques, and in 2009 they were acupuncture, biofeedback, nutritional counseling, and spiritual healing [18]. The second, by the United States Defense Health Agency (DHA), was more comprehensive and found a total of 275 “CAM programs,” comprising 11 modalities, collectively available at 120 MTFs (29%) in 2013. The most widely offered were acupuncture, chiropractic, and clinical nutrition therapy [4]. The third, by Williams et al. in 2016, assessed usage only of acupuncture, biofeedback, and chiropractic/osteopathic manipulation in the MHS from 2010 to 2015. This study showed 240 MTFs providing at least one of the three modalities during the study period. Of the recipients, 12.8% of active-duty service members received chiropractic/osteopathic manipulation, 1.9% received acupuncture, and 0.9% received biofeedback, for a total of 14.9% receiving at least one of these procedures during the study period [19].

In addition to these studies, a fact sheet distributed by the Defense Center of Excellence for Psychological Health and Traumatic Brain Injury (DCoE) within the DHA lists 15 “Complementary and Alternative Medicines (CAM), modalities, and interventions” which may be practiced particularly for psychological health or brain injury, including clinical indications for usage [9]. However, the fact sheet does not differentiate between usage in the MHS and the Veterans' Administration (VA), which are separately administered health systems, and does not provide indications for every listed intervention.

Taken together, these four sources describe 23 integrative therapies, combined into 15 modalities, available in the MHS. Of those, 11 are considered nonconventional according to the Cochrane Collaboration [20]. However, there is some disagreement in classification, such as Petri and Delgado's listing of TENS therapy as a nonconventional modality [18], though it is not considered “alternative” by the Cochrane Collaboration [20], or clinical nutrition therapy, which is generally considered conventional [24], listed as an integrative modality by the DHA [4]. A list of modalities available in the MHS, with their classifications and indications of use where available, is presented in Table 1.

### 3.2. Indications for Use

Surveys of IM usage in the MHS by Petri and Delgado [18] and by the Defense Health Agency [4] did not connect modalities directly with indications for usage. The survey by Williams et al. showed that 90.1% of chiropractic/osteopathic encounters and 53.9% of acupuncture encounters were for musculoskeletal and connective tissue disorders, with 33.8% of osteopathic and 25.8% of acupuncture encounters for other unspecified disorders of the back. In contrast, the greatest percentage of biofeedback encounters (34.3%) was for rehabilitation procedures generally associated with mental health [19]. The DCoE fact sheet provides clinical indications for several modalities including acupuncture, chiropractic, mindfulness meditation, and herbal supplements and nutrition but does not cover the full range of modalities listed in the MHS [9]. For those modalities, publicly available, peer-reviewed literature was used to provide information on general clinical indications. These sources are also listed in Table 1.

## 4. Discussion

### 4.1. Individual Modalities, Conditions, and Evidence

This section provides further discussion of the 15 IM modalities assessed within the MHS, to include prevalence, conventional versus nonconventional usage, clinical indications with particular focus on the polytrauma triad, and mechanism of action where known. These factors will provide a more complete picture of integrative modalities as used in the MHS.

*Acupuncture* has been consistently provided in the MHS since 2005 [18] and remains one of the most prevalent modalities, being provided at 83 MTFs in 2013 [4]. Acupuncture is of particular interest for the potential to relieve chronic pain without reliance on opioid drugs [25, 27, 70, 71]. Additionally, meta-analyses have found effectiveness of acupuncture to be greater than placebo in cases including back pain, knee pain, and headache [72]. Evidence is mixed but overall positive for treatment of PTSD and currently insufficient for treatment of TBI [28, 56, 73]. Techniques known in use within the MHS include dry needling, modified Chinese acupuncture, meridian-based French technique, scalp acupuncture, tendinomuscular acupuncture, moxibustion, and electrical stimulation [74–76]. Of particular interest in the MHS is an auricular technique known as “battlefield acupuncture,” in which small pins are placed into the ear, frequently to control pain [77, 78]. However, the overall prevalence of the separate techniques is not reported in literature.

The effectiveness of acupuncture is often attributed to the placebo effect; however, research has expanded our understanding of the mechanisms to include stimulation of somatic afferent nerves [79], differential modulation of neurotransmitters [80], and direct triggering of myofascial release points [81]. Functional MRI demonstrates differential activation of pain-controlling regions of the brain in verum versus sham acupuncture, suggesting that this change is not due entirely to the placebo effect [82, 83]. This is in line with the view of Western Medical Acupuncture, which focuses on localized neuromuscular effects [81] and does not consider itself “alternative” despite the Cochrane classification.

*Biofeedback* was provided at 13 MTFs in 2013 [4]. This technique involves sensors that allow the wearer to detect and learn to control functions that are normally autonomous. Systematic review indicates that biofeedback is not effective for treatment of PTSD [28] but appears to be effective overall for ameliorating psychiatric symptoms [32]. This is notable as psychosocial factors such as resilience have been shown to play a significant role in control of chronic pain [84–86]. Similarly, various biofeedback techniques appear to be effective for physical rehabilitation of neuromuscular and cardiovascular disorders [30]. One particular example is the Computer Assisted Rehabilitation Environment (CAREN), which provides visual feedback as part of a virtual-reality based training for rehabilitation, particularly in the case of limb loss [31]. While overall experience is positive, the environment can exacerbate issues such as motion sickness, and actual effectiveness varies on a case-by-case basis [31, 87].

*Breathing based therapy* was provided at 9 MTFs in 2013 [4]; however, as a component of many meditation and mindfulness techniques, it is likely more prevalent. This technique is often used in a mind-body setting in order to generate a relaxation response and frequently forms part of conventional behavioral interventions to address stress or anxiety. Recent studies suggest that breathing therapy works by reversing hypocapnia, a lower-than-normal concentration of carbon dioxide in the blood, and may be combined with biofeedback instruments to achieve this goal [33].

*Chiropractic* is another popular modality which has been provided in the MHS since at least 2005 [18] and was provided at 58 MTFs in 2013 [4]. This program of spinal manipulations (adjustments) is intended to correct small misalignments (often called subluxations) that contribute to pain and other disorders. This was originally thought to address systemic disease; however, modern understanding focuses on nonsurgical spinal healthcare [88], while chiropractors themselves acknowledge the limited evidence for subluxations and the divisiveness of the term [89]. This new focus recognizes the need for chiropractic to distinguish itself from other professions and establish scientific credibility [90]. Chiropractors subscribing to this new focus argue against the portrayal of their discipline as alternative [88], despite the Cochrane classification. While chiropractic and other forms of spinal manipulation are popular choices to address lower back pain, the evidence for lasting benefit is mixed. One Cochrane review concludes that certain types of spinal manipulation offer temporary relief but not greater relief than either sham manipulation or current therapies [91].

*Cognitive-behavioral therapies* (CBTs) were provided at 5 MTFs in 2013 [4]; however, as a standard set of tools in clinical psychology [34], the actual prevalence is likely much greater. These techniques focus on changing the patterns of thought in order to change behaviors or emotions associated with those thoughts [92]. CBTs are regarded as a “must try” for PTSD, though they seem to work best as early intervention for acute-onset symptoms, versus later intervention for chronic subacute symptoms, and effectiveness can vary between individuals [36, 37]. CBTs are also useful for the “unlearning” process in controlling chronic pain [35] and as an adjunct therapy for controlling anxiety and depression in TBI patients [38, 39]. Interestingly, CBTs show some effectiveness when provided by phone versus in person [36, 39], thus making therapy more convenient and avoiding much of the stigma associated with visiting a mental health provider.

*Hypnosis* was provided at 6 surveyed MTFs in 2005 and 2009 [18] but was not listed in the 2013 survey [4]. This modality may be loosely defined as the ability of a subject to respond to imaginative suggestions which are delivered following an appropriate induction ritual [93], though consensus definitions have varied through time and are subject to debate by the providers [94]. There is similar debate as to its effectiveness. A review by Wahbeh et al. concludes that hypnosis may be generally helpful for PTSD despite mixed results [28]; however, no review exists for management of TBI or for chronic pain in adults by hypnosis. A 2006 study in currently deployed personnel showed that a technique called Heart Centered Hypnotherapy (HCH) reduced symptoms of PTSD as measured by a standardized checklist [40]. However, the small sample size of 10 participants in each group is insufficient for general recommendations of this therapy.

*Light therapy* was provided at 4 surveyed MTFs in 2009 [18] and was not listed in the 2013 survey [4]. Per the Cochrane classification [20], usage may be either conventional or nonconventional. Exact usage within the MHS is not well-published. However, a pilot study of light therapy shows effectiveness for treating depression in military members [43] and has been suggested as a means of improving sleep for those working night shifts [95]. Growing evidence suggests that near-infrared laser treatment is effective for TBI [42], monochromatic infrared energy (MIRE) is effective for pain control [44–46], wound healing, and diabetic neuropathy [47–49], and chromotherapy is effective for treatment of neuropsychiatric disorders and wound healing [50–52]. However, these techniques are not specifically mentioned within the MHS, and further research is needed to determine the exact techniques in use, in order to permit full review of the evidence.

*Soft tissue manipulation* includes modalities such as massage, myofascial release, and structural integration (“Rolfing”). Massage is listed at 9 MTFs in 2013 [4]. Structural integration is not specifically listed in 2013 [4] and is listed at 3 surveyed MTFs in 2009 [18]. Massage is a standard technique of physical therapy, and evidence for its use is generally positive for pain relief in the general population but less so for pain relief in patients with cancer or following surgery [53, 54, 96]. Similarly, evidence for its effectiveness in low back pain and for control of tendonitis in the knee or elbow is mixed or inconclusive [25, 97, 98]. However, it remains generally popular among patients as well as those seeking relaxation. The specific technique of structural integration combines myofascial massage techniques with postural alignment in an effort to promote optimum physical and psychological health [99]. The massage component in particular may provide genuine medical benefit when considered as stretching of the fascia [100]. However, exact types of massage and specific indications for use within the MHS are not well reported in literature. Further research is necessary to determine this.

*Meditation, guided imagery, and mindfulness* are closely related techniques that may be used singly or in concert as either standalone therapies or adjuncts to conventional psychotherapy [101]. Within the MHS, meditation is reported at 14 MTFs in 2013 [4]. Imagery is reported at 7 surveyed MTFs in 2009 [18] and is not listed in the 2013 survey but may be included as part of meditation. A particular component of many meditation therapies is mindfulness, which promotes bringing of conscious attention to some internal factor (such as breathing). This allows the practitioner to become more aware of internal responses and to recognize “trigger” symptoms more quickly [102]. This may serve as a reciprocal activity to the stress inoculation practiced during combat training, which prepares warfighters to handle increased stress but not necessarily to calm down afterward [57]. A technique called Mindfulness-Based Mind Fitness Training (MMFT) was demonstrated in active-duty Marines to be effective at enabling calming responses as measured by heart rate, breathing, self-reported stress score, blood neuropeptides, and functional MRI, compared to the same measurements in Marines receiving training as usual [58]. The technique has been used to address both PTSD and TBI, though results as a standalone treatment are inconclusive for PTSD and mixed for TBI [55, 56]. Nonetheless, mindfulness forms an important and growing component of conventional medicine, and systematic review describes its effectiveness for controlling symptoms of both physical and psychological disorders [103–105].

*Naturopathic medicine*, as reported by the 2013 survey, is practiced at only one facility, Tripler Army Medical Center in Honolulu [4]. Naturopathic practitioners are commonly associated with primary care and make use of modalities such as homeopathy and botanical medicine which are not commonly provided in conventional medical care [59, 60]. Although naturopathic medical education requires a four-year postgraduate program in similar fashion to the requirements for MD and DO degrees, the focus on nonconventional modalities and the optional versus required residency [106] pose a challenge for acceptance by the wider medical community. The American Association of Family Physicians rejects naturopathic physicians' status as primary care doctors and strongly recommends against their licensure [107]. However, 17 states plus the District of Columbia, Puerto Rico, and US Virgin Islands do license naturopathic physicians, while Massachusetts and Pennsylvania expect licensure in 2017 and 2018, respectively [108]. In this capacity, naturopathic physicians may perform diagnostic tests and minor surgery, administer injectable and IV therapies, prescribe drugs, and refer patients to specialists [109]; however, specific medical privileges vary by state. The exact indications for which naturopathy is provided within the MHS are not reported in literature.

*Nutrition* includes both conventional and nonconventional usage, though highly variable terminology hampers this distinction. Clinical nutrition is reported as an integrative modality in use at 68 MTFs in 2013 [4], while “nutritional counseling” is reported at 12 surveyed MTFs in 2009 [18]. The conventional and nonconventional focus on avoidance of chronic disease are similar. However, the integrative modality takes particular account of psychosocial factors, stresses personalized plans, and works to transition patients away from the standard American diet (SAD) and avoid toxins [61]. A related area is the use of (nonbotanical) supplements, which again may be conventional or nonconventional depending on usage [20]. The exact usage of integrative nutrition within the MHS is not well reported. However, dietary and protein supplements are often sought by military personnel for real or perceived wellness and for performance enhancement [110, 111].

*Spiritual healing* was reported at 3 MTFs in 2013 [4] and 12 surveyed MTFs in 2009 [18]. Depending on the backgrounds of the practitioner and the person seeking care, the practice may include anything from standard chaplain services to laying on of hands, distance healing, or energy manipulation [62], none of which are in conventional medical usage. Spiritual prayer may also form an important foundation of psychotherapies, particularly those aimed at resolving survivor's guilt and other sources of internal conflict [63]. It may also be used as a component of another modality, such as yoga or meditation [112].

*Transcutaneous Electrical Nerve Stimulation (TENS)* is reported at 9 surveyed MTFs in 2009 [18] and is not reported on the 2013 survey [4]. This procedure involves applying a small, targeted electric current to an area of the body in order to relieve pain. TENS is routinely performed as part of conventional physical therapy and has a clear mechanism of action described in both the central and peripheral components of the nervous system [113]. Despite this, evidence for effectiveness is mixed. The Cochrane reviews show insufficient evidence to recommend TENS for low back pain [114] and to evaluate this therapy for chronic pain due to missing info and small sample sizes in the studies surveyed [115]. Other confounding issues include study heterogeneity, inadequate blinding of providers, cooccurrence of other therapies, and location of treatment sites [114, 116]. Overall, this picture of conflicting evidence highlights the need for more studies.

*Yoga* is reported at 4 surveyed MTFs in 2009 [18] and is not listed in the 2013 survey [4]. This discipline combines gentle movement and stretching with breathing exercises and meditation, though focus on particular components can vary between practitioners and disciplines. Several studies have shown effectiveness of yoga in a military setting. A 2012 study showed that daily yoga helps troops in combat to build physiologic resilience, similar to the effect demonstrated for mindfulness above [67]. Trauma-sensitive yoga, especially a technique called iRest, works this way to draw attention inward and focus on the parasympathetic nervous system and shows promise for addressing symptoms of PTSD [117]. Similarly, the spiritual or meditative component of yoga can help to address anxiety, depression, and schizophrenia [112]. Methods of action include reduction of stress hormones cortisol and ACTH [118] and increase of GABA [119]. A systematic review and meta-analysis showed that yoga was strongly effective in the short term and moderately effective in the long term for relieving chronic lower back pain; however, results were not significantly different from those observed for other forms of exercise [66]. Yoga also shows promise for increasing balance and reducing fall risk after stroke and improving function following TBI [68, 69]. However, the specific traditions and techniques of yoga provided within the MHS are not well-described in literature.

### 4.2. Overall Themes

Three themes became apparent during the course of this review. First, the principles of IM are well-suited to address the multifactorial issues underlying the polytrauma triad. Chronic pain, TBI, and PTSD can mutually reinforce one another in a cycle that is difficult to break and which poses a challenge for conventional medicine [10, 11]. The mind-body connection in particular is well-established for the polytrauma triad, and literature shows significant relief of symptoms brought about by certain mind-body interventions [26, 120–122]. Based on this review, specific, effective interventions to address the polytrauma triad include mindfulness (which may include breathing techniques), CBT, acupuncture, chiropractic, and yoga. These are among the modalities practiced by innovative residential programs such as those at the National Intrepid Center for Excellence (NICoE) [123] and the Cincinnati VA Medical Center [124]. Notably, chiropractic and acupuncture are among the most widely provided integrative modalities at MTFs [4, 19], which is consistent with a focus on the relief of chronic pain.

Second, as mentioned in the introduction, much of the push for IM comes from the service members themselves. A large military cohort study (*n* = 44,287) showed 39% of respondents using nonconventional therapies over the course of a year, while such usage was correlated with greater number of health conditions compared to those who used solely conventional therapies [7]. Similarly, another study shows 30% of military respondents (*n* = 86,131) using at least one practitioner-assisted, nonconventional therapy during a three-year period, with such usage again correlated with a greater number of health conditions [17]. Chronic pain in particular is a major driver of nonconventional therapy usage [125]. Efforts to incorporate such therapies into the MHS stem from recognition of existing patterns of usage and a desire to provide more nonopioid pain management options for service members [125]. However, it should be noted that members may also receive care through civilian facilities under the TRICARE insurance plan, which generally does not pay for treatments considered “alternative” [126]. Therefore, such care must be paid out of pocket if it cannot be acquired at an MTF.

Third, research continues to illustrate new mechanisms of action for some modalities, which in turn builds the evidence base for continuing research. Incorporation of nonconventional modalities into mainstream medicine has previously provoked a rift between those who propose to build the evidence base for such treatments and to allow more provider and patient leeway in choosing them [3, 127] and those who oppose such investigation and usage without prior establishment of biological plausibility [128]. However, modern understanding of biological mechanisms supports prior plausibility for many modalities. For instance, acupuncture originally posited the idea of energy flow along meridians [81]; chiropractic posited vertebral subluxations as a cause of systemic disease [129]; and structural integration posited an alignment of the body's energy field with Earth's gravity [99]. In contrast, modern literature describes acupuncture in terms of localized neuromuscular effects and neurotransmitter release [81], chiropractic emphasizes its focus on spinal health [90], and proper postural alignment is a fundamental tenet of modern ergonomics [130]. Similarly, the placebo effect, which is thought to underlie the success of many integrative modalities, is now understood in terms of differential neurotransmitter and brain region activity shown on a functional MRI [131, 132]. This notably includes activation of the endogenous opioid system, along with other mechanisms described by continuing research [133–135]. Such changes in understanding establish plausible means of action for these modalities and collectively provide support for continued research into nonconventional therapies.

## 5. Limitations of Review

This review has two significant limitations. First, as military providers are not incentivized to publish, there is a paucity of literature discussing the use of IM specifically within the MHS. Second, the major surveys of IM in the MHS do not connect modalities directly with diagnoses or clinical indications and are insufficiently granular to differentiate between techniques practiced within a single modality (e.g., the type of acupuncture or yoga). Although some information is available through other sources, connecting the modalities with practice in a single document is important for establishing overall prevalence as well as determining conventional versus nonconventional usage. Taken together, these factors hamper a full review of the evidence.

## 6. Conclusion

This review highlights the wide range of IM options available within the MHS. Given the patient-driven demand for these modalities, their potential for addressing chronic disease in an effective and coordinated way, and a new focus on promoting access to these therapies, it is likely that the scope of IM in the MHS will continue expanding. Further research, such as secondary analysis of healthcare claims, will help to illustrate the larger picture of IM in the MHS and to continue building the evidence base for these treatments. This full picture will help to inform sound medical, psychological, and financial decision-making to provide the best and most cost-effective care to military members and their families.

## Figures and Tables

**Table 1:** Integrative modalities in the Military Health System.

Modality	# of MTFs (2013) [4]	Conventional? [20]	Clinical indications	Notes
Acupuncture [4, 9, 18, 19]	83	No	Chronic pain, stress, anxiety, TBI, substance withdrawal [9], chronic pain [9, 25–27], PTSD [9, 28, 29]	

Biofeedback [4, 9, 18, 19]	13	No	Physical rehabilitation [30, 31], mental health issues [32]	

Breath-based practices [4, 9]	9	No	Relaxation, reversal of hypocapnia [33]	Often a component of meditation or yoga

Chiropractic [4, 9, 18, 19]	58	No	Pain in back or neck, headache, radiculopathy [9]	
Osseous manipulation [9, 19]				

Cognitive behavioral therapy [4]	5	Yes	Psychological issues [34], chronic pain [35], PTSD [36, 37], TBI, anxiety, depression [38, 39]	

Hypnosis [18]	Not listed	No	PTSD [28, 40, 41], TBI [41]	

Light therapy [18]	Not listed	Varies	TBI [42], depression [43], pain control [44–46], wound healing [47–52], neuropsychiatric disorders [50–52]	Multiple techniques; exact uses in MHS not established in literature

Massage [4, 18]	9	No	Relaxation, physical therapy, sports medicine, pain control [53, 54]	
Soft tissue manipulation [9]				
Structural integration [18]	3			

Meditation [4, 9, 18]	14	No	Stress management, anxiety, PTSD, TBI [55, 56], pain, depression [9], relaxation, control of anxiety [57, 58]	Mostly for mindfulness
Meditative behavioral techniques [18] Imagery [18] Mindfulness [9]				

Naturopathy [4, 9]	1	No	Primary care [59, 60]	

Nutritional counseling [18]	Not listed	Varies	Hypertension, diabetes, weight loss, coronary disease, prevention, general health [9], prevention of chronic disease [61]	“Diet therapy” listed as alternative by Cochrane but specific usage may vary
Clinical nutrition [4, 9]	68			

Relaxation [18]	Not listed	No	May be provided through massage, breathing techniques, or meditation (see modalities)	Listed as conventional for PTSD [29]

Spiritual healing [18]	3	No	Relaxation, psychotherapy, resolution of internal conflict [62, 63]	

TENS [18]	Not listed	Yes	Pain control [64, 65]	Not listed alternative by Cochrane

Yoga [4, 9, 18]	11	No	Relaxation, pain control [66], building of mental resilience [67], rehabilitation [68, 69]	
Movement meditation [9]				

## References

[1] American Board of Physician Specialties (2017). *Integrative Medicine Defined*.

[2] National Center for Complementary and Integrative Health (2016). Complementary, Alternative, or Integrative Health: What’s In a Name?. *NCCIH*.

[3] Institute of Medicine (US) Committee on the Use of Complementary and Alternative Medicine by the American Public (2005). *Complementary and Alternative Medicine in the United States*.

[4] U.S. Defense Health Agency (DHA) (2014). *Integrative Medicine in the Military Health System: Report to Congress*.

[5] Cassileth B. R., Vickers A. J. (2005). High prevalence of complementary and alternative medicine use among cancer patients: Implications for research and clinical care. *Journal of Clinical Oncology*.

[6] Esteghamati A., Mazaheri T., Vahidi Rad M., Noshad S. (2015). Complementary and alternative medicine for the treatment of obesity: a critical review. *International Journal of Endocrinology and Metabolism*.

[7] White M. R., Jacobson I. G., Smith B. (2011). Health care utilization among complementary and alternative medicine users in a large military cohort. *BMC Complementary and Alternative Medicine*.

[8] Cifu D. X., Taylor B. C., Carne W. F. (2013). Traumatic brain injury, posttraumatic stress disorder, and pain diagnoses in OIF/OEF/OND Veterans. *Journal of Rehabilitation Research and Development*.

[9] Defense Centers of Excellence for Psychological Health and Traumatic Brain Injury. Complementary and Alternative Medicines (CAM), Modalities, and Interventions

[10] Lew H. L. (2009). Prevalence of chronic pain, posttraumatic stress disorder, and persistent postconcussive symptoms in OIF/OEF veterans: polytrauma clinical triad. *Journal of Rehabilitation Research and Development (JRRD)*.

[11] Otis J. D., Keane T. M., Kerns R. D. (2003). An examination of the relationship between chronic pain and post-traumatic stress disorder. *Journal of Rehabilitation Research and Development*.

[12] Jonas W. B., Schoomaker E. B. (2014). Pain and opioids in the military: we must do better. *JAMA Internal Medicine*.

[13] Butler L. D., Linn B. K., Meeker M. A., McClain-Meeder K., Nochajski T. H. (2015). We Don't Complain About Little Things': Views of Veterans and Military Family Members on Health Care Gaps and Needs. *Military Behavioral Health*.

[14] Goertz C., Marriott B. P., Finch M. D. (2013). Military report more complementary and alternative medicine use than civilians. *The Journal of Alternative and Complementary Medicine*.

[15] Prince P. D. (2011). Army medicine officials seek to evolve warrior care. *The United States Army*.

[16] McPherson F., Schwenka M. A. (2004). Use of complementary and alternative therapies among active duty soldiers, military retirees, and family members at a military hospital. *Military Medicine*.

[17] Jacobson I. G., White M. R., Smith T. C. (2009). Self-Reported Health Symptoms and Conditions Among Complementary and Alternative Medicine Users in a Large Military Cohort. *Annals of Epidemiology*.

[18] Petri R. P., Delgado R. E. (2015). Integrative Medicine Experience in the U.S. Department of Defense. *Med. Acupunct*.

[19] Williams V. F., Clark L. L., McNellis M. G. (2016). Use of complementary health approaches at military treatment facilities, active component, U.S. Armed Forces, 2010-2015. *MSMR*.

[20] Wieland L. S., Manheimer E., Berman B. M. (2011). Development and classification of an operational definition of complementary and alternative medicine for the Cochrane collaboration. *Alternative Therapies In Health And Medicine*.

[21] White House Commission on Complementary and Alternative Medicine Policy. White House Commission on Complementary and Alternative Medicine Policy Final Report.

[22] About Us - Number of Beneficiaries, TRICARE. http://www.tricare.mil/About/Facts/BeneNumbers.

[23] About Us - Health and Dental Facilities, TRICARE. http://www.tricare.mil/About/Facts/Facilities.

[24] Cederholm T., Barazzoni R., Austin P. (2017). ESPEN guidelines on definitions and terminology of clinical nutrition. *Clinical Nutrition*.

[25] Furlan A. D., Giraldo M., Baskwill A., Irvin E., Imamura M. (2015). *Cochrane Database of Systematic Reviews*.

[26] Lee C., Crawford C., Hickey A., Active Self-Care Therapies for Pain (PACT) Working Group (2014). Mind-body therapies for the self-management of chronic pain symptoms. *Pain Med. Malden Mass*.

[27] Trinh K., Graham N., Irnich D., Cameron I. D., Forget M. (2016). *Cochrane Database of Systematic Reviews*.

[28] Wahbeh H., Senders A., Neuendorf R., Cayton J. (2014). Complementary and Alternative Medicine for Posttraumatic Stress Disorder Symptoms: A Systematic Review. *Journal of Evidence-Based Complementary and Alternative Medicine*.

[29] Libby D. J., Pilver C. E., Desai R. (2012). Complementary and alternative medicine in VA specialized PTSD Treatment Programs. *Psychiatric Services*.

[30] Giggins O. M., Persson U. M., Caulfield B. (2013). Biofeedback in rehabilitation. *J. Neuro Engineering Rehabil*.

[31] Isaacson B. M., Swanson T. M., Pasquina P. F. (2013). The use of a computer-assisted research environment (CAREN) for enhancing wounded warrior rehabilitation regimens. *Journal of Spinal Cord Medicine*.

[32] Schoenberg P. L. A., David A. S. (2014). Biofeedback for psychiatric disorders: a systematic review. *Appl. Psychophysiol. Biofeedback*.

[33] Meuret A. E., Rosenfield D., Seidel A., Bhaskara L., Hofmann S. G. (2010). Respiratory and cognitive mediators of treatment change in panic disorder: evidence for intervention specificity. *Journal of Consulting and Clinical Psychology*.

[34] Hofmann S. G., Asnaani A., Vonk I. J. J., Sawyer A. T., Fang A. (2012). The efficacy of cognitive behavioral therapy: a review of meta-analyses. *Cogn. Ther. Res*.

[35] Shpaner M., Kelly C., Lieberman G. (2014). Unlearning chronic pain: A randomized controlled trial to investigate changes in intrinsic brain connectivity following Cognitive Behavioral Therapy. *NeuroImage: Clinical*.

[36] Stecker T., McHugo G., Xie H., Whyman K., Jones M. (2014). A randomized controlled trial of a phone-based cognitive-behavioral intervention to improve PTSD treatment utilization among returning service members. *Psychiatr. Serv. Wash DC*.

[37] Qi W., Gevonden M., Shalev A. (2016). Prevention of Post-Traumatic Stress Disorder After Trauma: Current Evidence and Future Directions. *Current Psychiatry Reports*.

[38] Hsieh M.-Y., Ponsford J., Wong D., McKay A. (2012). Exploring variables associated with change in cognitive behaviour therapy (CBT) for anxiety following traumatic brain injury. *Disability and Rehabilitation*.

[39] Fann J. R., Bombardier C. H., Vannoy S. (2015). Telephone and in-person cognitive behavioral therapy for major depression after traumatic brain injury: A randomized controlled trial. *Journal of Neurotrauma*.

[40] Yarvis J. S. (2008). Hypnotherapy under fire: efficacy of heart-centered hynotherapy in the treatment of iraq war veterans with posttraumatic stress. *J. Heart-Centered Ther*.

[41] McDonough V. T., Queen E. (2014). How Do You Use Hypnotherapy to Help Patient with TBI and/or PTSD?. *Brainlinemilitary*.

[42] Morries L. D., Cassano P., Henderson T. A. (2015). Treatments for traumatic brain injury with emphasis on transcranial near-infrared laser phototherapy. *Neuropsychiatric Disease and Treatment*.

[43] Lande R. G., Williams L. B., Gragnani C., Tsai A. (2011). Effectiveness of light therapy for depression among active duty service members: a nonrandomized controlled pilot trial. *Complement. Ther. Med*.

[44] Kuan T.-S., Lin Y.-C., Lien W.-C. (2015). The effect of monochromatic infrared photo energy on the irritability of myofascial trigger spot of rabbit skeletal muscle. *Evidence-based Complementary and Alternative Medicine*.

[45] Ammar T. A. R. A. (2015). Monochromatic infrared photo energy versus low level laser therapy in chronic low back pain. *Journal of Lasers in Medical Sciences*.

[46] Hsieh R.-L., Lo M.-T., Lee W.-C., Liao W.-C. (2012). Therapeutic effects of short-term monochromatic infrared energy therapy on patients with knee osteoarthritis: a double-blind, randomized, placebo-controlled study. *J. Orthop. Sports Phys. Ther*.

[47] Franzen-Korzendorfer H., Blackinton M., Rone-Adams S., McCulloch J. (2008). The effect of monochromatic infrared energy on transcutaneous oxygen measurements and protective sensation: Results of a controlled, double-blind, randomized clinical study. *Ostomy Wound Management*.

[48] He Y., Yip S. L. Y., Cheung K.-K., Huang L., Wang S., Cheing G. L. Y. (2013). The effect of monochromatic infrared energy on diabetic wound healing. *International Wound Journal*.

[49] Powell M. W., Carnegie D. E., Burke T. J. (2004). Reversal of diabetic peripheral neuropathy and new wound incidence: the role of MIRE. *Advance in Skin & Wound Care*.

[50] Azeemi S. T. Y., Raza S. M. (2005). A critical analysis of chromotherapy and its scientific evolution. *Evid. Based Complement. Alternat. Med*.

[51] Fushimi T., Inui S., Nakajima T., Ogasawara M., Hosokawa K., Itami S. (2012). Green light emitting diodes accelerate wound healing: characterization of the effect and its molecular basis in vitro and in vivo. *Wound Repair and Regeneration*.

[52] Adamskaya N., Dungel P., Mittermayr R. (2011). Light therapy by blue LED improves wound healing in an excision model in rats. *Injury*.

[53] Boyd C., Crawford C., Paat C. F., Price A., Xenakis L., Zhang W. (2016). The Impact of Massage Therapy on Function in Pain Populations—A Systematic Review and Meta-Analysis of Randomized Controlled Trials: Part II, Cancer Pain Populations. *Pain Medicine*.

[54] Crawford C., Boyd C., Paat C. F. (2016). The Impact of Massage Therapy on Function in Pain Populations—A Systematic Review and Meta-Analysis of Randomized Controlled Trials: Part I, Patients Experiencing Pain in the General Population. *Pain Medicine*.

[55] Cukor J., Spitalnick J., Difede J., Rizzo A., Rothbaum B. O. (2009). Emerging treatments for PTSD. *Clinical Psychology Review*.

[56] Cantor J. B., Gumber S. (2013). Use of complementary and alternative medicine in treating individuals with traumatic brain injury. *Curr. Phys. Med. Rehabil. Rep*.

[57] Brewer J. (2014). Mindfulness in the military. *American Journal of Psychiatry*.

[58] Johnson D. C. (2014). Modifying resilience mechanisms in atrisk individuals: a controlled study of mindfulness training in Marines preparing for deployment. *American Journal of Psychiatry*.

[59] Fleming S. A., Gutknecht N. C. (2010). Naturopathy and the primary care practice. *Prim. Care Clin. Off. Pract*.

[60] Litchy A. P. (2011). Naturopathic physicians: Holistic primary care and integrative medicine specialists. *Journal of Dietary Supplements*.

[61] DeBusk R., Sierpina V. S., Kreitzer M. J. (2011). Applying functional nutrition for chronic disease prevention and management: Bridging nutrition and functional medicine in 21st century healthcare. *Explore: The Journal of Science and Healing*.

[62] Ernst E. (2006). Spiritual Healing: More Than Meets the Eye. *Journal of Pain and Symptom Management*.

[63] Kopacz M. S., Connery A. L., Bishop T. M. (2016). Moral injury: a new challenge for complementary and alternative medicine. *Complementary Therapies in Medicine*.

[64] Dailey D. L., Rakel B. A., Vance C. G. T. (2013). Transcutaneous electrical nerve stimulation reduces pain, fatigue and hyperalgesia while restoring central inhibition in primary fibromyalgia. *Pain*.

[65] Johnson M. I., Paley C. A., Howe T. E., Sluka K. A., The Cochrane Collaboration (2015). *Cochrane Database of Systematic Reviews*.

[66] Cramer H., Lauche R., Haller H., Dobos G. (2013). A systematic review and meta-analysis of yoga for low back pain. *The Clinical Journal of Pain*.

[67] Stoller C. C., Greuel J. H., Cimini L. S., Fowler M. S., Koomar J. A. (2012). Effects of sensory-enhanced yoga on symptoms of combat stress in deployed military personnel. *American Journal of Occupational Therapy*.

[68] Schmid A. A., Van Puymbroeck M., Altenburger P. A. (2012). Poststroke balance improves with yoga: a pilot study. *Stroke*.

[69] Schmid A. A., Miller K. K., Van Puymbroeck M., Schalk N. (2016). Feasibility and results of a case study of yoga to improve physical functioning in people with chronic traumatic brain injury. *Disability and Rehabilitation*.

[70] Lee M. S., Ernst E. (2011). Acupuncture for pain: an overview of cochrane reviews. *Chin. J. Integr. Med*.

[71] Linde K. (2016). *Cochrane Database of Systematic Reviews*.

[72] Hopton A., MacPherson H. (2010). Acupuncture for chronic pain: is acupuncture more than an effective placebo? A systematic review of pooled data from meta-analyses. *Pain Practice*.

[73] Wong V., Cheuk D. K., Lee S., Chu V. (2013). *Cochrane Database of Systematic Reviews*.

[74] Lee C., Wallerstedt D., Duncan A. (2011). Design and rationale of a comparative effectiveness study to evaluate two acupuncture methods for the treatment of headaches associated with traumatic brain injury. *Medical Acupuncture*.

[75] Niemtzow R. C., Gambel J., Helms J., Pock A., Burns S., Baxter J. (2006). Integrating ear and scalp acupuncture techniques into the care of blast-injured United States Military Service members with limb loss. *Journal of Alternative and Complementary Medicine*.

[76] Spira A. (2008). Acupuncture: A useful tool for health care in an operational medicine environment. *Military Medicine*.

[77] King H. C., Hickey A. H., Connelly C. (2013). Auricular acupuncture: A brief introduction for military providers. *Military Medicine*.

[78] Goertz C. M. H., Niemtzow R., Burns S. M., Fritts M. J., Crawford C. C., Jonas W. B. (2006). Auricular acupuncture in the treatment of acute pain syndromes: a pilot study. *Military Medicine*.

[79] Takahashi T. (2011). Mechanism of acupuncture on neuromodulation in the gut—a review. *Neuromodulation*.

[80] Chen S., Wang S., Rong P. (2014). Acupuncture for visceral pain: neural substrates and potential mechanisms. *Evidence-Based Complementary and Alternative Medicine*.

[81] White A. (2009). Editorial Board of Acupuncture in Medicine. Western medical acupuncture: a definition. *Acupunct. Med. J. Br. Med. Acupunct. Soc*.

[82] Usichenko T. I., Wesolowski T., Lotze M. (2015). Verum and sham acupuncture exert distinct cerebral activation in pain processing areas: a crossover fMRI investigation in healthy volunteers. *Brain Imaging and Behavior*.

[83] Egorova N., Gollub R. L., Kong J. (2015). Repeated verum but not placebo acupuncture normalizes connectivity in brain regions dysregulated in chronic pain. *NeuroImage: Clinical*.

[84] Newton-John T., Mason C., Hunter M. (2014). The role of resilience in adjustment and coping with chronic pain. *Rehabil. Psychol*.

[85] Alschuler K. N., Kratz A. L., Ehde D. M. (2016). Resilience and vulnerability in individuals with chronic pain and physical disability. *Rehabil. Psychol*.

[86] Ruiz-Párraga G. T., López-Martínez A. E., Esteve R., Ramírez-Maestre C., Wagnild G. (2015). A confirmatory factor analysis of the Resilience Scale adapted to chronic pain (RS-18): new empirical evidence of the protective role of resilience on pain adjustment. *Qual. Life Res. Int. J. Qual. Life Asp. Treat. Care Rehabil*.

[87] Highland K. B., Kruger S. E., Roy M. J. (2015). If You Build It, They Will Come, But What Will Wounded Warriors Experience? Presence in the CAREN. *Stud. Health Technol. Inform*.

[88] Brown R. (2012). A health care system in transformation: making the case for chiropractic. *Chiropractic and Manual Therapies*.

[89] Mirtz T. A., Perle S. M. (2011). The prevalence of the term subluxation in North American English-Language Doctor of chiropractic programs. *Chiropr. Man. Ther*.

[90] Nelson C. F. (2005). Chiropractic as spine care: a model for the profession. *Chiropr. Osteopat*.

[91] Rubinstein S. M., Terwee C. B., Assendelft W. J., de Boer M. R., van Tulder M. W. (2012). *Cochrane Database of Systematic Reviews*.

[92] National Association of Cognitive-Behavioral Therapists. What is Cognitive-Behavioral Therapy (CBT)? National Association of Cognitive-Behavioral Therapists.

[93] Lynn S. J., Laurence J.-R., Kirsch I. (2015). Hypnosis, suggestion, and suggestibility: an integrative model. *Am. J. Clin. Hypn*.

[94] Lynn S. J., Green J. P., Kirsch I. (2015). Grounding Hypnosis in Science: The “New” APA Division 30 Definition of Hypnosis as a Step Backward. *American Journal of Clinical Hypnosis*.

[95] Caldwell J. L., Gilreath S. R. (2001). Work and sleep hours of U.S. Army aviation personnel working reverse cycle. *Military Medicine*.

[96] Boyd C., Crawford C., Paat C. F., Price A., Xenakis L., Zhang W. (2016). The Impact of Massage Therapy on Function in Pain Populations—A Systematic Review and Meta-Analysis of Randomized Controlled Trials: Part III, Surgical Pain Populations. *Pain Medicine*.

[97] Hernandez-Reif M., Field T., Krasnegor J., Theakston H. (2001). Lower back pain is reduced and range of motion increased after massage therapy. *International Journal of Neuroscience*.

[98] Loew L. M. (2014). *Cochrane Database of Systematic Reviews*.

[99] Jacobson E. (2011). Structural integration: origins and development. *Journal of Alternative and Complementary Medicine*.

[100] Grimm D. (2007). Cell biology meets rolfing. *Science*.

[101] Holmes E. A., Mathews A. (2010). Mental imagery in emotion and emotional disorders. *Clin. Psychol. Rev*.

[102] Malinowski P. (2013). Neural mechanisms of attentional control in mindfulness meditation. *Frontiers in Neuroscience*.

[103] Creswell J. D. (2017). Mindfulness Interventions. *Annual Review of Psychology*.

[104] Dimidjian S., Segal Z. V. (2015). Prospects for a clinical science of mindfulness-based intervention. *American Psychologist*.

[105] Gotink R. A., Chu P., Busschbach J. J. V., Benson H., Fricchione G. L., Hunink M. G. M. (2015). Standardised mindfulness-based interventions in healthcare: An overview of systematic reviews and meta-analyses of RCTs. *PLoS ONE*.

[106] Post-Graduate Naturopathic Residencies, AANMC. https://aanmc.org/naturopathic-residencies/.

[107] American Association of Family Physicians. Naturopathic Practice. http://www.aafp.org.

[108] Association of Accredited Naturopathic Colleges. Naturopathic Doctor Licensure | List of States and Provinces. AANMC. https://aanmc.org/resources/licensure/.

[109] AANP - American Association of Naturopathic Physicians: Natural Medicine. Real Solutions. http://www.naturopathic.org/.

[110] Austin K. G., Price L. L., McGraw S. M., Lieberman H. R. (2015). Predictors of dietary supplement use by U.S. Coast Guard personnel. *PLoS ONE*.

[111] Lieberman H. R., Stavinoha T. B., McGraw S. M., White A., Hadden L. S., Marriott B. P. (2010). Use of dietary supplements among active-duty US Army soldiers. *American Journal of Clinical Nutrition*.

[112] Varambally S., Gangadhar B. N. (2012). Yoga: A spiritual practice with therapeutic value in psychiatry. *Asian Journal of Psychiatry*.

[113] Vance C. G. T., Dailey D. L., Rakel B. A., Sluka K. A. (2014). Using TENS for pain control: the state of the evidence. *Pain management*.

[114] Khadilkar A., Odebiyi D. O., Brosseau L., Wells G. A., The Cochrane Collaboration (2008). Cochrane Database of Systematic Reviews.

[115] Nnoaham K., Kumbang J. (2008). Transcutaneous electrical nerve stimulation (TENS) for chronic pain. *Cochrane Database Syst Rev*.

[116] DeSantana J. M., Walsh D. M., Vance C., Rakel B. A., Sluka K. A. (2008). Effectiveness of Transcutaneous Electrical Nerve Stimulation for Treatment of Hyperalgesia. *Current Rheumatology Reports*.

[117] Stankovic L. (2011). Transforming trauma: a qualitative feasibility study of integrative restoration (iRest) yoga Nidra on combat-related post-traumatic stress disorder.. *International journal of yoga therapy*.

[118] Vedamurthachar A., Janakiramaiah N., Hegde J. M. (2006). Antidepressant efficacy and hormonal effects of Sudarshana Kriya Yoga (SKY) in alcohol dependent individuals. *Journal of Affective Disorders*.

[119] Streeter C. C., Jensen J. E., Perlmutter R. M. (2007). Yoga Asana sessions increase brain GABA levels: a pilot study. *Journal of Alternative and Complementary Medicine*.

[120] Kim S. H., Schneider S. M., Kravitz L., Mermier C., Burge M. R. (2013). Mind-body Practices for Posttraumatic Stress Disorder. *J. Investig. Med. Off. Publ. Am. Fed. Clin. Res*.

[121] Cosio D., Swaroop S. (2016). The use of mind-body medicine in chronic pain management: differential trends and session-by-session changes in anxiety. *J. Pain Manag. Med*.

[122] Bédard M. (2012). Mindfulness-based cognitive therapy: benefits in reducing depression following a traumatic brain injury. *Adv. Mind Body Med*.

[123] Foote F. O., Schwartz L. (2012). Holism at the National Intrepid Center of Excellence (NICoE). *Explore: The Journal of Science and Healing*.

[124] Hernández T. D., Brenner L. A., Walter K. H., Bormann J. E., Johansson B. (2016). Complementary and alternative medicine (CAM) following traumatic brain injury (TBI): Opportunities and challenges. *Brain Research*.

[125] Office of the Army Surgeon General. Pain Management Task Force Report: Providing a Standardized DoD and VHA Vision and Approach to Pain Management to Optimize the Care for Warriors and their Families.

[126] Defense Health Agency. Exclusions, TRICARE. http://www.tricare.mil/CoveredServices/IsItCovered/Exclusions.

[127] Rutten L., Stolper E. (2015). Complementary alternative medicine, plausibility and statistics. *Eur. J. Intern. Med*.

[128] Pandolfi M., Carreras G. (2014). The faulty statistics of complementary alternative medicine (CAM). *European Journal of Internal Medicine*.

[129] Mirtz T. A., Morgan L., Wyatt L. H., Greene L. (2009). An epidemiological examination of the subluxation construct using Hills criteria of causation. *Chiropr. Osteopat*.

[130] Claus A. P., Hides J. A., Moseley G. L., Hodges P. W. (2016). Thoracic and lumbar posture behaviour in sitting tasks and standing: progressing the biomechanics from observations to measurements. *Appl. Ergon*.

[131] Diederich N. J., Goetz C. G. (2008). The placebo treatments in neurosciences: New insights from clinical and neuroimaging studies. *Neurology*.

[132] Lidstone S. C., Stoessl A. J. (2007). Understanding the placebo effect: contributions from neuroimaging. *Mol. Imaging Biol*.

[133] Benedetti F. (2008). Mechanisms of placebo and placebo-related effects across diseases and treatments. *Annual Review of Pharmacology and Toxicology*.

[134] Eippert F., Bingel U., Schoell E. D. (2009). Activation of the opioidergic descending pain control system underlies placebo analgesia. *Neuron*.

[135] Enck P., Benedetti F., Schedlowski M. (2008). New Insights into the Placebo and Nocebo Responses. *Neuron*.

